# People as solutions to sustainability

**DOI:** 10.1007/s13280-024-02070-z

**Published:** 2024-08-30

**Authors:** Karen O’Brien, Gail Hochachka

**Affiliations:** 1 Department of Sociology and Human Geography, University of Oslo, Blindern, P.O. Box 1096, 0317 Oslo, Norway; 2 Department of Wood Science, Faculty of Forestry, 2424 Main Mall, University of British Columbia, Vancouver, BC V6T 1Z4 Canada

Comment to: Yttredal, E.R., J. Löffler, and K.M. Tschorn. 2024. From the question how to act in a sustainable manner, back to the question why we act unsustainably. *Ambio*. 10.1007/s13280-024-02024-5.

We appreciate the opportunity to respond to Yttredal et al.’s (2024) commentary on “Fractal Approaches to Scaling Transformations to Sustainability” (O’Brien et al. 2023). Their concern is that the concept of universal values overlooks how social priorities have evolved to work against sustainability principles, proposing that our social brains may be a root cause of unsustainable development. Interestingly, we interpret this research differently and conclude that humans can also be considered the solution to sustainability challenges. Below, we elaborate on why our social brains may be key to sustainability and how universal values can help connect us to others and to principles such as environmental integrity, social justice, and interconnectedness.

The question of why people do not choose sustainable alternatives, even when available, has been a focus of research in social sciences and humanities. While psychological research shows that our brains are “wired to connect,” the nature and quality of human actions and interactions are dynamic and context-dependent, influenced by innovations, institutions, infrastructure, and policies, as well as interests and power relationships. These factors can nudge people toward unsustainable behaviors, but they can also foster political agency leading to structural changes for collective well-being.

Research from evolutionary theory, emerging neuroscience, and human value theories suggests that our social brains can contribute significantly to solving sustainability challenges. For example, Lieberman (2013) emphasizes that we have the ability to reflect on our characteristics, beliefs, and values in relation to others, and to use self-control to pursue long-term goals. Siegel (2023) discusses how we can “construct the most integrative experience of self [that is] possible to promote well-being in our world.” Similarly, Schlitz et al. (2010) highlighted the relationship between worldview transformations and social consciousness, noting that awareness of being part of a larger, interconnected community is key to sustainability.

Adult development research shows that mindsets and meaning-making can evolve over a lifetime to embrace greater complexity and more perspectives (Kegan & Lahey 2009). Research also indicates that more complex mindsets are better suited to addressing global issues like climate change and thus have unique contributions to make in sustainability solutions (Lynam 2012; Hochachka 2022). Worldviews that recognize meaning, value, or consciousness correlate positively with connectedness to nature and pro-environmental attitudes and lifestyles (Hedlund-de Witt et al. 2014). Deep connections with nature are inherent in many worldviews and cosmologies, including many Indigenous worldviews.

The fractal approach described in our paper draws attention to relational paradigms, emphasizing integration of the practical, political, and personal spheres of transformation. We highlight how certain values, such as equity, dignity, and compassion, can apply universally and generate self-similar patterns that repeat across scales. While values are diverse, contextual, and associated with different motivations, universal values relate to everyone, i.e., to the whole.

Yttredal et al. (2024) raise the important question of whether universal values are normative or can be considered a fundamental law of nature. Some researchers suggest that universal values are crucial precursors to global well-being and sustainable development. For example, the UN considers universal values as dynamic constructs that are a normative practice (Lang 2020). Others argue that these values are part of what it means to be human, and that all persons share some morally significant commonalities (Caney 2006). Some extend these values to animals and nature, recognizing their inherent right to flourish (Nussbaum 2024).

Fractal approaches to sustainability emphasize the potential for universal values to replicate across contexts and scales. Practicing “fractal agency” based on universal values fosters connections that transcend polarization and fragmentation. Recognizing that we are “intraconnected” parts of a larger whole, our social minds can work *for* rather than against sustainability. As Siegel (2023, 109) writes, “[w]e survive by honoring the interdependent nature of our reality, not by ignoring this fundamentally connected essence of who we are.”
